# The effect of a single *SMARCA4* exon deletion on RNA splicing: Implications for variant classification

**DOI:** 10.1002/mgg3.2232

**Published:** 2023-07-10

**Authors:** Anna Byrjalsen, Ulrik Stoltze, Mana Mehrjouy, Jane Hübertz Frederiksen, Mads Bak, Ulf Birkedal, Henrik Hasle, Anne‐Marie Gerdes, Kjeld Schmiegelow, Karin Wadt, Thomas van Overeem Hansen

**Affiliations:** ^1^ Department of Clinical Genetics, Rigshospitalet Copenhagen University Hospital Copenhagen Denmark; ^2^ Department of Pediatrics and Adolescent Medicine, Rigshospitalet Copenhagen University Hospital Copenhagen Denmark; ^3^ Department of Pediatrics Aarhus University Hospital Aarhus N Denmark; ^4^ Department of Clinical Medicine, Faculty of Health and Medical Sciences University of Copenhagen Copenhagen Denmark

**Keywords:** de novo, single‐exon deletion, small cell carcinoma of the ovary, *SMARCA4*, splicing, variant classification

## Abstract

**Background:**

Exon deletions are generally considered pathogenic, particularly when they are located out of frame. Here, we describe a pediatric, female patient presenting with hypercalcemia and a small cell carcinoma of the ovary, hypercalcemic type, and carrying a germline *de novo* *SMARCA4* exon 14 deletion.

**Methods:**

The *SMARCA4* deletion was identified by whole genome sequencing, and the effect on the RNA level was examined by gel‐ and capillary electrophoresis and nanopore sequencing.

**Results:**

The deletion was in silico predicted to be truncating, but RNA analysis revealed two major transcripts with deletion of exon 14 alone or exon 14 through 15, where the latter was located in‐frame. Because the patient's phenotype matched that of other patients with pathogenic germline variants in *SMARCA4*, the deletion was classified as likely pathogenic.

**Conclusion:**

We propose to include RNA analysis in classification of single‐exon deletions, especially if located outside of known functional domains, as this can identify any disparate effects on the RNA and DNA level, which may have implications for variant classification using the American College of Medical Genetics and Genomics guidelines.

## Graphical abstract

We present a pediatric, female patient presenting with hypercalcemia and a small cell carcinoma of the ovary and carrying a germline de novo *SMARCA4* deletion of exon 14. The deletion was in silico predicted to be truncating, but RNA analysis generated two major transcripts with deletion of either exon 14 alone or both exon 14 and 15, where the latter was in‐frame. Because the patient phenotype matched that of other patients with pathogenic germline variants in *SMARCA4*, the deletion was classified as likely pathogenic.

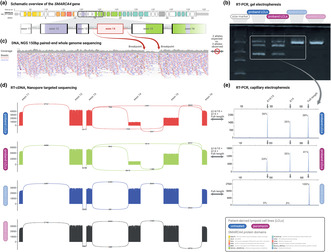

## 1 INTRODUCTION

The *SMARCA4* gene is a chromatin regulator operating within the SWI/SNF pathway (Mardinian et al., 2021). Pathogenic somatic variants in the genes of the SWI/SNF pathway occur in 20% of human malignancy (Hodges et al., 2016). Pathogenic truncating germline variants in *SMARCA4* (OMIM #603254) cause rhabdoid tumor predisposition syndrome (RTPS) type 2 (OMIM #613325; Connor et al., 2021), which is characterized by an increased lifetime risk of developing atypical teratoid/rhabdoid tumor in the central nervous system (CNS), rhabdoid tumors outside of CNS, and small cell carcinoma of the ovary hypercalcemic type (SCCOHT), whereas missense variants or small in‐frame deletions within the highly conserved ATPase/helicase domain of *SMARCA4*, predispose to Coffin‐Siris syndrome (CSS), characterized by developmental delay, microcephaly, and facial anomalies (Mardinian et al., 2021). Pathogenic truncating variants in *SMARCA4* have been considered predominantly inherited from a parent with the condition (Connor et al., 2021; Holsten et al., 2018; Pastorczak et al., 2021; Schneppenheim et al., 2010).

Evaluation of genetic variants is based on gene‐specific consensus guidelines (Richards et al., 2015; Riggs et al., 2020). Exon deletions are—in accordance with these guidelines—classified as pathogenic when generating a truncating frameshift in a gene with a known disease phenotype caused by loss‐of‐function (LoF) variants.

Here, we present a 9‐year‐old girl with an ovarian tumor, who carried a germline *de novo* exon 14 deletion in *SMARCA4*, which showed a differential effect on the RNA level. This unexpected finding indicates that RNA analysis of single‐exon deletions should be performed routinely to ensure correct variant classification.

## 2 MATERIALS AND METHODS

### 2.1 Ethical compliance

Ethical approval was obtained through the regional scientific ethical committee (the Ethical Scientific Committees for the Capital Region, H‐15016782) and the Danish Data Protection Agency (RH‐2016‐219, I‐Suite no: 04804).

### 2.2 Whole genome sequencing

Whole genome sequencing (WGS) and data analysis were performed as recently described elsewhere (Byrjalsen et al., 2020). The NM 001128849.3 transcript was used to describe the *SMARCA4* deletion at the transcript level, since this transcript is the reference transcript used in the Human Gene Mutation Database variant database (https://digitalinsights.qiagen.com/products‐overview/clinical‐insights‐portfolio/human‐gene‐mutation‐database/). However, at least nine alternative *SMARCA4* transcripts have been described, including the MANE (NM_003072.5) and MANE Plus Clinical (NM_001387283.1) transcripts, where the identified genomic *SMARCA4* deletion also affects exon 14. However, in other *SMARCA4* transcripts, the genomic deletion affects either exon 13 (e.g., NM_001128845.2) or exon 15 (NM_001128844.3).

### 2.3 Samples

Heparinized blood samples from the patient were used for generation of lymphoblastoid cell lines (LCLs) by Epstein–Barr virus immortalization of B lymphocytes employing standard procedures. The cells were maintained in Bioamf‐3‐complete media in a humidified atmosphere of 5% at 37°C. LCLs were treated with puromycin (250 μg/mL) for 4 h to inhibit nonsense‐mediated decay (NMD) when indicated.

### 2.4 RNA purification

RNA was purified from LCLs using the Chemagic Total RNA Kit H24 (Chemagen), on a chemagic 360 (Chemagen) according to the manufacturer's instructions. The RNA concentration and quality were determined using the Agilent RNA 6000 Nano Kit (Agilent) and 2100 Bioanalyzer (Agilent).

### 2.5 RNA analysis

cDNA was generated using 500 ng of RNA, a mixture of random hexamer and oligo‐dT template primers, and the SuperScript IV First‐Strand Synthesis System (#18091050, Invitrogen) as recommended by the manufacturer. Polymerase chain reaction (PCR) Primers were designed to target exon 11; SMARCA4_Ex11F 5′‐AGAAGGACAGACGCCTGCCATTGG‐3′ and exon 16; SMARCA4_Ex16R 5′‐CCGTTCAGGTTGTTGTTGTACAGG‐3′ following recommendations to amplify at least one whole exon up‐ or downstream of the variant (Whiley et al., 2014). The PCR reaction was performed in 30‐μL reaction containing 5 μL of cDNA using HotStarTaq DNA polymerase (#203205, Qiagen) and the following PCR program: 95°C for 10 min, followed by 35 cycles consisting of 95°C for 1 min, 60°C for 1 min, and 72°C for 1 min, and an extension step at 72°C for 7 min. RT‐PCR products from the patient sample and at least two control samples were analyzed by agarose gel electrophoresis and purified using QIAquick Gel Extraction kit (#28704, Qiagen). The RT‐PCR products were also analyzed by capillary electrophoresis using primers labeled with FAM at the 5′‐end. RT‐PCR products were diluted 1:20 or 1:40 and analyzed on an ABI3730 Genetic Analyzer (Applied Biosystems) using the following conditions: temperature 66°C, 3 s injection at 2.0 kV, and 1400 s run at 15 kV. GeneScan LIZ‐500 and ROX‐1000 were used as internal size standards depending on the estimated size of the RT‐PCR products. Fragments were analyzed by GeneMapper software v6.0 (Applied Biosystems). Only peaks between 150 relative fluorescent units (RFUs) and 10,000 RFUs were included for data analysis. The splicing fraction was calculated for each transcript by dividing the peak area of the individual transcript with the sum of all peak areas (all transcripts) as recently described (Montalban et al., 2019).

### 2.6 Nanopore cDNA sequencing and data analysis

Barcoded nanopore sequencing libraries were made with 67.9 ng (corresponding to 200 fmol) of each sample and control amplicon as input, using Native Barcoding Kit 24 (SQK‐NBD112.24) and the Ligation sequencing amplicons protocol version NBA_9135_v112_revF_01Dec2021 from Oxford Nanopore Technologies (Oxford). The libraries were sequenced along with 20 other barcoded libraries on a MinION sequencer with a FLO‐MIN106 flow cell which was run for 1 h and 20 m and operated using MinKNOW v. 22.03.6. Basecalling and demultiplexing were handled in real time by MinKNOW using the fast model; res_dna_r9.4.1_e8.1_fast_v033. The resulting sequence reads were mapped to the human genome GRCh38 using Minimap2 (Li, 2018) v. 2.17‐r941 with parameters for splice awareness, −ax splice and best map output only, and −secondary = no. Full length reads (with coverage of both exon 11 and 16) were selected using Samtools v1.6. Sashimi plots showing coverage and exon junction counts were made with ggsashimi (Garrido‐Martín et al., 2018) modified in‐house to take in sample‐specific exon junction count cut‐off, set to 5% of the read count.

## 3 RESULTS

**FIGURE 1:**
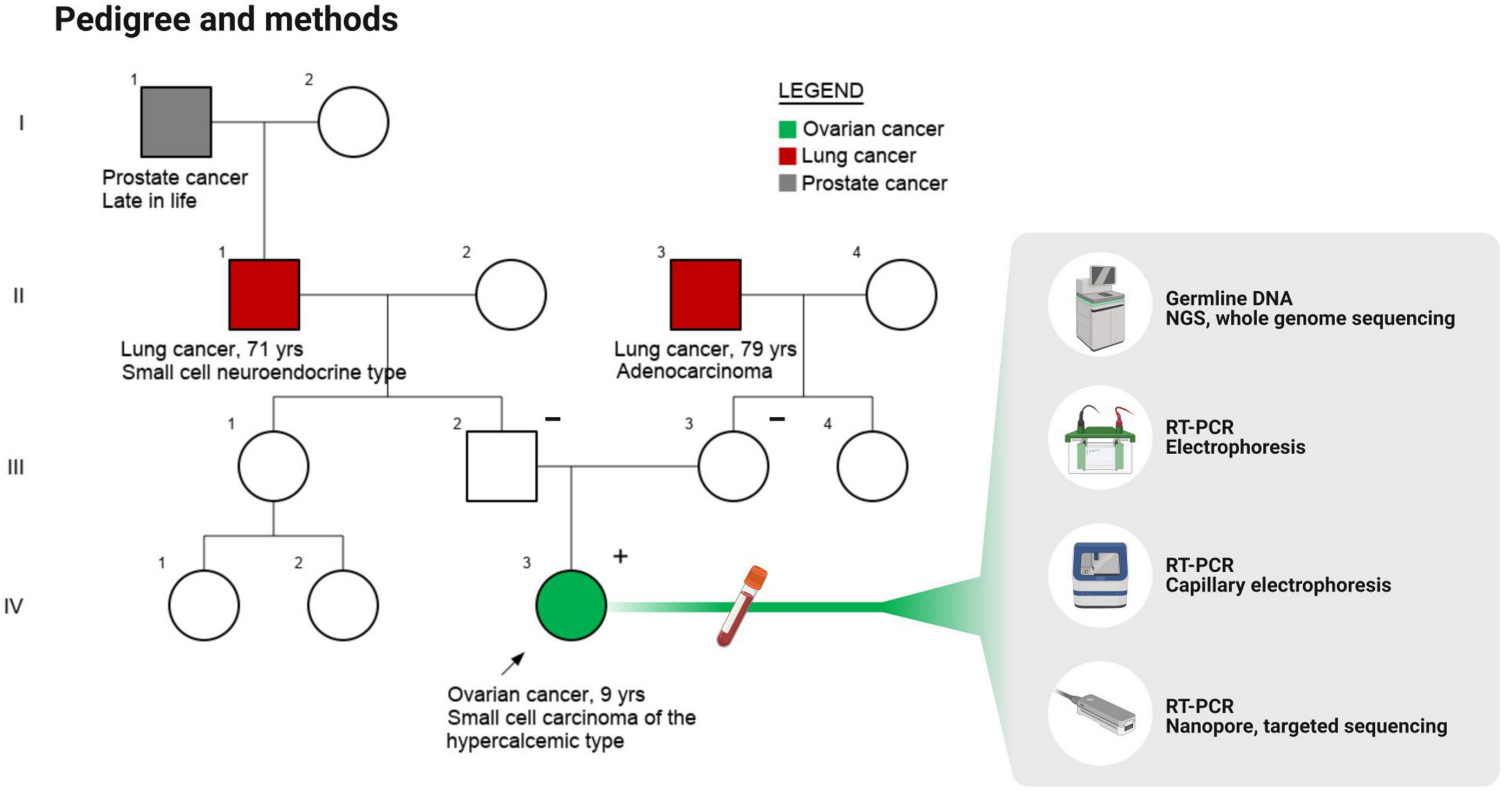
Family pedigree and methodology. The left side of the figure shows the family pedigree. The arrow indicates the index patient. Circles: women. Squares: men. The right side of the figure shows the testing performed on heparinized blood from the index patient. Starting with germline whole genome sequencing, followed by gel‐ and capillary electrophoresis and nanopore sequencing.

A previously healthy 9‐year‐old girl was hospitalized after 10 days of nausea and vomiting. Initial blood work revealed total calcium levels of 4.1 mmol/L (ref 2.3–2.7), ionized‐Ca^2+^ levels of 2.4 mmol/L (ref 1.2–1.3), and phosphate levels of 0.7 mmol/L (ref 1.2–1.8). Blood pressure was 130/100 mm Hg, and the echocardiogram showed sinus rhythm. In addition, creatinine levels were elevated (78 μmol/L, ref 34–62). Treatment with bisphosphonate, fluids, and furosemide normalized calcium, creatinine, and the blood pressure. Ultrasound and magnetic resonance imaging (MRI) revealed a tumor of 7 × 6.5 × 10 cm in the right ovary. Positron Emissions Tomography and Computed Tomography showed no metastases. The tumor, right ovary, and adnexa underwent complete surgical resection. Initial pathology report classified the tumor as an anaplastic synovial sarcoma, and the patient was treated with four courses of ifosfamide and doxorubicin. The left ovary was removed for cryopreservation. The patient remains in complete remission with 50 months of follow‐up. The pedigree did not reveal other cancers except for smoking‐related lung cancers in a maternal and a paternal grandfather later in life (Figure 1).

The patient was offered WGS as part of the STAGING study, a nation‐wide WGS study offered to all patients newly diagnosed with pediatric cancer in Denmark from 2017 and onward (Byrjalsen et al., 2020). WGS revealed a germline 2.1 kb deletion including exon 14 of the *SMARCA4* gene (NG_011556.3, GRCh38, chr19:11,007,277‐11,009,336, NM 001128849.3) predicted to result in a frameshift and a premature stop codon (c.2002‐625_2124‐1045del, p.(Glu669Cysfs*7)). The *SMARCA4* deletion was not identified in the patient's parents, confirming that the variant arose *de novo*. The variant has not previously been reported in the medical literature, gnomAD, Decipher or ClinVar. Upon reevaluation of the tumor, the pathologist found that the tumor was a malignant rhabdoid tumor corresponding to a SCCOHT.

**FIGURE 2:**
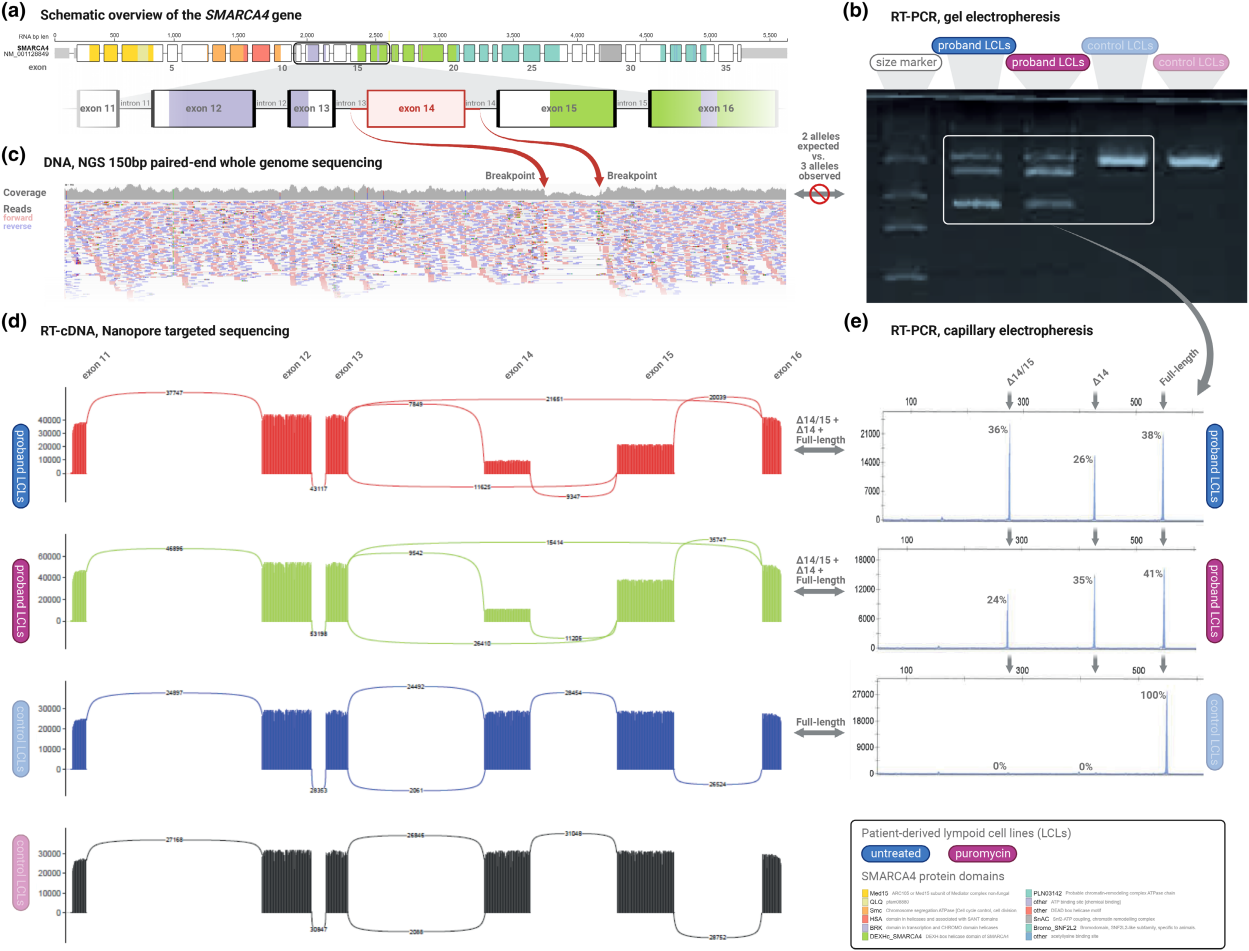
Sequencing results. (a) Showing a schematic visualization of the *SMARCA4* gene and an enlargement of the area covering the deletion of exon 14. (b) Picture of the gel electrophoresis analysis of the RT‐PCR product obtained from untreated LCLs from the *SMARCA4* exon 14 deletion carrier, puromycin‐treated LCLs from the *SMARCA4* exon 14 deletion carrier as well as control LCLs. (c) Visualization of the paired‐end reads from the whole genome sequencing showing the deletion and the reduction of reads in this region. (d) Sashimi plots of nanopore cDNA sequencing performed on RT‐PCR products obtained from untreated LCLs from the *SMARCA4* exon 14 deletion carrier, puromycin‐treated LCLs from the *SMARCA4* exon 14 deletion carrier as well as control LCLs. (e) Capillary electrophoresis analysis of the RT‐PCR product obtained from untreated LCLs from the *SMARCA4* exon 14 deletion carrier, puromycin‐treated LCLs from the *SMARCA4* exon 14 deletion carrier as well as control LCLs.

To verify the deletion and to assess its functional effect, heparin tubes for RNA analyses were collected from the patient. RNA was extracted from lymphoblasts treated or untreated with puromycin, and PCR was performed with primers targeting exon 11 and exon 16. The RT‐PCR products were then analyzed by gel and capillary electrophoresis as well as nanopore cDNA (Figure 2). The analysis identified two major alternative transcripts in the patient sample, not present in the control samples. Capillary electrophorese and nanopore sequencing revealed that the alternative transcripts lacked exon 14 and exon 14─15, respectively. Although skipping of exon 14 introduced a frameshift and a premature stop codon (p.Glu669Cysfs*7), skipping of exon 14─15 lead to an in‐frame deletion of 91 amino acids (668─758). Using capillary electrophorese data, quantification of the splicing events in puromycin‐treated cells indicated that approximately 35% of the transcripts had skipping of exon 14, while approximately 23% had skipping of exon 14─15. Since no informative heterozygote single nucleotide polymorphism (SNP) was present in the *SMARCA4* transcript, allele‐specific expression analysis was not performed.

## 4 DISCUSSION

Guidelines for evaluation of potential pathogenicity of novel genetic variation are continuously being developed to aid and unify variant classification (Richards et al., 2015). Richards et al. (2015) recommend dividing variants into categories based on criteria assessing the evidence of pathogenicity: Very strong (PSV1), Strong (PS1─4), Moderate (PM1─6), or Supporting (PP1─5). Single or multiexon deletion, which introduces a frameshift, is considered PSV1 when occurring in a gene where LoF is a known disease mechanism, although care should be taken not to overinterpret variants (e.g., when located in cold‐spot regions). These criteria can then be compiled (if the variant fulfills more than one criterion) and used for categorization as pathogenic, likely pathogenic, variant of unknown significance (VUS), likely benign or benign. In our case, at first glance, the variant fulfills PSV1 at the DNA level as the variant is predicted to introduce a frameshift and a premature stop codon. As the variant was not reported in any population databases or in the literature, the criteria PM2 supporting is also applied, and the variant was classified as likely pathogenic (class 4). This corresponds to two exon 14 acceptor site splice variants (c.2002‐1G > A and c.2002‐2A > G), which are also classified as likely pathogenic in ClinVar (accession number VCV001525165.4 and VCV000583116.3).

In order to validate the DNA finding, we performed RNA analysis. Surprisingly, the RNA analysis revealed two major alternative transcripts, one in accordance with the predicted effect on the DNA level and one resulting in an in‐frame skipping of exon 14─15 deleting 91 amino acids in an area without any known functional domains, located between the BRK domain, which is suggested to be involved in protein–protein interactions (Allen et al., 2020), and the ATPase/helicase domain. In isolation, this in‐frame deletion which removes less than 10% of the coding sequence would be downgraded to PSV1 supporting according to some gene‐specific American College of Medical Genetics and Genomics guidelines leading to classification of VUS (class 3). Quantification analysis by capillary electrophorese revealed that approximately 35% of the transcripts from the patient sample had truncating skipping of exon 14, while approximately 23% had skipping of exon 14─15, causing an in‐frame deletion. The phenomenon is well known from canonical splicing variants, which can also result in deletion of several exons (Anna & Monika, 2018). Even though two different transcripts (truncating and in‐frame deletion) are expressed, the patient described in this study only presented with the somewhat milder phenotype SCCOHT disease and not CSS. Previously, one patient with both SCCOHT and mild CSS was described; however, this patient had a nonsense variant in exon 19 of the *SMARCA4* gene (Errichiello et al., 2017). Currently, we do not know the functional effect of the in‐frame deletion caused by exon 14 and 15 skipping, and we can therefore not regard this as a rescue transcript.

Of note, analysis of very large aggregations of whole exome/genome sequencing data has revealed that some genes in the human gene pool have significantly fewer LoF variants than expected (Karczewski et al., 2020). Such genes are termed constrained, and monoallelic LoF variants within these are understood to have been under tremendous evolutionary pressure. Recently, genes, in which monoallelic LoF variant cause an increased risk of cancer in childhood, were shown to exhibit significant constraint (Stoltze et al., 2022). *SMARCA4* was among the most constrained genes (LoF rate was 1% of expected, CI 90%: 0%–6%), suggesting that LoF variants in *SMARCA4* are under selective pressure. The fact that the variant in *SMARCA4* occurred *de novo* supports this theory. Inherited LoF *SMARCA4* variants have been reported in the literature a number of times, whereas *SMARCA4* *de novo* variants have only been reported twice (Errichiello et al., 2017; Tischkowitz et al., 2020; Witkowski et al., 2013).

Roughly a fourth of carriers of variants in *SMARCA4* are unaffected, rendering the criteria related to inheritance less relevant when evaluating a variant in *SMARCA4* (Holsten et al., 2018). As the deletion identified in our case fits with the phenotype, this specific variant is classified as likely pathogenic (class 4), but this may not always be the case. We would therefore propose that classification of a single‐exon deletion should be supported by functional tests assessing the effect on the RNA level before the deletion may confidently be deemed disease causing.

## 5 CONCLUSION

Based on these findings, we recommend that truncating single‐exon deletions, but also multi‐exon deletions, are verified on the RNA level before they are classified as PVS1, unless other features as deletion size, localization in functional domains, and/or the phenotype strongly suggest pathogenicity. Further, larger RNA studies of single‐exon deletions are necessary to determine the frequency of our finding.

## AUTHOR CONTRIBUTIONS

Thomas van Overeem Hansen identified the case and came up with the concept of the paper, Anna Byrjalsen wrote the initial draft, Ulrik Stoltze drafted the figures, Mana Mehrjouy, Jane Hübertz Frederiksen, Mads Bak, and Ulf Birkedal analyzed genomic and functional data. Henrik Hasle were responsible for the research–clinical interaction with the family. Anna Byrjalsen, Ulrik Stoltze, Mana Mehrjouy, Jane Hübertz Frederiksen, Mads Bak, Ulf Birkedal, Henrik Hasle, Anne‐Marie Gerdes, Kjeld Schmiegelow, Karin Wadt, and Thomas van Overeem Hansen revised the manuscript. Anna Byrjalsen submitted the manuscript.

## FUNDING INFORMATION

The study (as part of the STAGING study) was funded by the Danish Childhood Cancer Foundation, Region Hovedstaden's Foundation for Health Research, the Neye Foundation, and Engineer Otto Christensen's Foundation.

## CONFLICT OF INTEREST STATEMENT

To the best of our knowledge, no conflicts of interest, financial or otherwise, exists.

## ETHICS STATEMENT

Ethical approval was obtained through the regional scientific ethical committee (the Ethical Scientific Committees for the Capital Region, H‐15016782) and the Danish Data Protection Agency (RH‐2016‐219, I‐Suite no: 04804).

## Data Availability

The data presented in this paper can be made available from the corresponding author upon reasonable request.

## References

[mgg32232-bib-0001] Allen, M. D. , Bycroft, M. , & Zinzalla, G. (2020). Structure of the BRK domain of the SWI/SNF chromatin remodeling complex subunit BRG1 reveals a potential role in protein–protein interactions. Protein Science, 29(4), 1047–1053. 31909846 10.1002/pro.3820 PMC7096718

[mgg32232-bib-0002] Anna, A. , & Monika, G. (2018). Splicing mutations in human genetic disorders: Examples, detection, and confirmation. Journal of Applied Genetics, 59(3), 253–268. 29680930 10.1007/s13353-018-0444-7 PMC6060985

[mgg32232-bib-0003] Byrjalsen, A. , Hansen, T. V. O. , Stoltze, U. K. , Mehrjouy, M. M. , Barnkob, N. M. , Hjalgrim, L. L. , Mathiasen, R. , Lautrup, C. K. , Gregersen, P. A. , Hasle, H. , Wehner, P. S. , Tuckuviene, R. , Sackett, P. W. , Laspiur, A. O. , Rossing, M. , Marvig, R. L. , Tommerup, N. , Olsen, T. E. , Scheie, D. , … Wadt, K. (2020). Nationwide germline whole genome sequencing of 198 consecutive pediatric cancer patients reveals a high frequency of cancer prone syndromes. PLoS Genetics, 16(12), 1–24. 10.1371/journal.pgen.1009231 PMC7787686 33332384

[mgg32232-bib-0004] Connor, Y. D. , Miao, D. , Lin, D. I. , Hayne, C. , Howitt, B. E. , Dalrymple, J. L. , DeLeonardis, K. R. , Hacker, M. R. , Esselen, K. M. , & Shea, M. (2021). Germline mutations of SMARCA4 in small cell carcinoma of the ovary, hypercalcemic type and in SMARCA4‐deficient undifferentiated uterine sarcoma: Clinical features of a single family and comparison of large cohorts. Gynecologic Oncology, 157(1), 106–114. 10.1016/j.ygyno.2019.10.031 PMC7887697 31954538

[mgg32232-bib-0005] Errichiello, E. , Mustafa, N. , Vetro, A. , Notarangelo, L. D. , de Jonge, H. , Rinaldi, B. , Vergani, D. , Giglio, S. R. , Morbini, P. , & Zuffardi, O. (2017). SMARCA4 inactivating mutations cause concomitant Coffin–Siris syndrome, microphthalmia and small‐cell carcinoma of the ovary hypercalcaemic type. The Journal of Pathology, 243(1), 9–15. 28608987 10.1002/path.4926 PMC5601212

[mgg32232-bib-0006] Garrido‐Martín, D. , Palumbo, E. , Guigó, R. , & Breschi, A. (2018). ggsashimi: Sashimi plot revised for browser‐ and annotation‐independent splicing visualization. PLoS Computational Biology, 14(8), 1–6. 10.1371/journal.pcbi.1006360 PMC6114895 30118475

[mgg32232-bib-0007] Hodges, C. , Kirkland, J. G. , & Crabtree, G. R. (2016). Complexes in cancer (pp. 1–24). Cold Spring Harb Lab Press. 10.1101/cshperspect.a026930 PMC4968166 27413115

[mgg32232-bib-0008] Holsten, T. , Bens, S. , Oyen, F. , Nemes, K. , Hasselblatt, M. , Kordes, U. , Siebert, R. , Frühwald, M. C. , Schneppenheim, R. , & Schüller, U. (2018). Germline variants in SMARCB1 and other members of the BAF chromatin‐remodeling complex across human disease entities: A meta‐analysis. European Journal of Human Genetics, 26(8), 1083–1093. 10.1038/s41431-018-0143-1 29706634 PMC6057970

[mgg32232-bib-0009] Karczewski, K. J. , Francioli, L. C. , Tiao, G. , Cummings, B. B. , Alföldi, J. , Wang, Q. , Collins, R. L. , Laricchia, K. M. , Ganna, A. , Birnbaum, D. P. , Gauthier, L. D. , Brand, H. , Solomonson, M. , Watts, N. A. , Rhodes, D. , Singer‐Berk, M. , England, E. M. , Seaby, E. G. , Kosmicki, J. A. , … MacArthur, D. G. (2020). The mutational constraint spectrum quantified from variation in 141,456 humans. Nature, 581(7809), 434–443. 32461654 10.1038/s41586-020-2308-7 PMC7334197

[mgg32232-bib-0010] Li, H. (2018). Minimap2: Pairwise alignment for nucleotide sequences. Bioinformatics, 34(18), 3094–3100. 29750242 10.1093/bioinformatics/bty191 PMC6137996

[mgg32232-bib-0011] Mardinian, K. , Adashek, J. J. , Botta, G. P. , Kato, S. , & Kurzrock, R. (2021). SMARCA4: Implications of an altered chromatin‐remodeling gene for cancer development and therapy. Molecular Cancer Therapeutics, 20(12), 2341–2351. 34642211 10.1158/1535-7163.MCT-21-0433 PMC8643328

[mgg32232-bib-0012] Montalban, G. , Bonache, S. , Moles‐Fernández, A. , Gadea, N. , Tenés, A. , Torres‐Esquius, S. , Carrasco, E. , Balmaña, J. , Diez, O. , & Gutiérrez‐Enríquez, S. (2019). Incorporation of semi‐quantitative analysis of splicing alterations for the clinical interpretation of variants in BRCA1 and BRCA2 genes. Human Mutation, 40(12), 2296–2317. 31343793 10.1002/humu.23882

[mgg32232-bib-0013] Pastorczak, A. , Krajewska, K. , Urbanska, Z. , Szmyd, B. , Salacinska‐Los, E. , Kobos, J. , Mlynarski, W. , & Trelinska, J. (2021). Ovarian carcinoma in children with constitutional mutation of SMARCA4: Single‐family report and literature review. Family Cancer, 20(4), 355–362. 10.1007/s10689-021-00258-w PMC8484133 33907931

[mgg32232-bib-0014] Richards, S. , Aziz, N. , Bale, S. , Bick, D. , das, S. , Gastier‐Foster, J. , Grody, W. W. , Hegde, M. , Lyon, E. , Spector, E. , Voelkerding, K. , Rehm, H. L. , & ACMG Laboratory Quality Assurance Committee . (2015). Standards and guidelines for the interpretation of sequence variants: A joint consensus recommendation of the American College of Medical Genetics and Genomics and the Association for Molecular Pathology. Genetics in Medicine, 17(5), 405–424. 25741868 10.1038/gim.2015.30 PMC4544753

[mgg32232-bib-0015] Riggs, E. R. , Andersen, E. F. , Cherry, A. M. , Kantarci, S. , Kearney, H. , Patel, A. , Raca, G. , Ritter, D. I. , South, S. T. , Thorland, E. C. , Pineda‐Alvarez, D. , Aradhya, S. , & Martin, C. L. (2020). Technical standards for the interpretation and reporting of constitutional copy‐number variants: A joint consensus recommendation of the American College of Medical Genetics and Genomics (ACMG) and the clinical genome resource (ClinGen). Genetic Medicine, 22(2), 245–257. 10.1038/s41436-019-0686-8 PMC7313390 31690835

[mgg32232-bib-0016] Schneppenheim, R. , Frühwald, M. C. , Gesk, S. , Hasselblatt, M. , Jeibmann, A. , Kordes, U. , Kreuz, M. , Leuschner, I. , Subero, J. I. M. , Obser, T. , Oyen, F. , Vater, I. , & Siebert, R. (2010). Germline nonsense mutation and somatic inactivation of SMARCA4/BRG1 in a family with rhabdoid tumor predisposition syndrome. American Journal of Human Genetics, 86(2), 279–284. 20137775 10.1016/j.ajhg.2010.01.013 PMC2820190

[mgg32232-bib-0017] Stoltze, U. K. , Foss‐Skiftesvik, J. , Hansen, T. , Byrjalsen, A. , Sehested, A. , Scheie, D. , Stamm Mikkelsen, T. , Rasmussen, S. , Bak, M. , Okkels, H. , Thude Callesen, M. , Skjøth‐Rasmussen, J. , Gerdes, A. M. , Schmiegelow, K. , Mathiasen, R. , & Wadt, K. (2022). Genetic predisposition & evolutionary traces of pediatric cancer risk: A prospective 5‐year population‐based genome sequencing study of children with CNS tumors. Neuro‐Oncology, 25, 761–773. 10.1093/neuonc/noac187 PMC10076945 35902210

[mgg32232-bib-0018] Tischkowitz, M. , Huang, S. , Banerjee, S. , Hague, J. , Hendricks, W. P. D. , Huntsman, D. G. , Lang, J. D. , Orlando, K. A. , Oza, A. M. , Pautier, P. , Ray‐Coquard, I. , Trent, J. M. , Witcher, M. , Witkowski, L. , McCluggage, W. G. , Levine, D. A. , Foulkes, W. D. , & Weissman, B. E. (2020). Small‐cell carcinoma of the ovary, hypercalcemic type‐genetics, new treatment targets, and current management guidelines. Clinical Cancer Research, 26(15), 3908–3917. 32156746 10.1158/1078-0432.CCR-19-3797 PMC7415570

[mgg32232-bib-0019] Whiley, P. J. , de la Hoya, M. , Thomassen, M. , Becker, A. , Brandão, R. , Pedersen, I. S. , Montagna, M. , Menéndez, M. , Quiles, F. , Gutiérrez‐Enríquez, S. , de Leeneer, K. , Tenés, A. , Montalban, G. , Tserpelis, D. , Yoshimatsu, T. , Tirapo, C. , Raponi, M. , Caldes, T. , Blanco, A. , … ENIGMA consortium . (2014). Comparison of mRNA splicing assay protocols across mulitple laboratories: Recommendations for best practice in standardized clinical testing. Clinical Chemistry, 60(2), 341–352. 24212087 10.1373/clinchem.2013.210658 PMC4351044

[mgg32232-bib-0020] Witkowski, L. , Lalonde, E. , Zhang, J. , Albrecht, S. , Hamel, N. , Cavallone, L. , May, S. T. , Nicholson, J. C. , Coleman, N. , Murray, M. J. , Tauber, P. F. , Huntsman, D. G. , Schönberger, S. , Yandell, D. , Hasselblatt, M. , Tischkowitz, M. D. , Majewski, J. , & Foulkes, W. D. (2013). Familial rhabdoid tumour “avant la lettre”—From pathology review to exome sequencing and back again. The Journal of Pathology, 231(1), 35–43. 23775540 10.1002/path.4225

